# The left ventricular “lining” technique for repair of ischemic ventricular septal rupture

**DOI:** 10.1007/s11748-023-01994-9

**Published:** 2023-12-18

**Authors:** Takahiro Katsumata, Ryo Shimada, Hiroaki Uchida, Tatsuya Suzuki, Hideki Ozawa, Masahiro Daimon

**Affiliations:** Department of Thoracic and Cardiovascular Surgery, Osaka Medical and Pharmaceutical University, 2-7 Daigaku-machi, Takatsuki, Osaka 569-8686 Japan

**Keywords:** Ventricular septal rupture, Left ventricular rupture, Myocardial infarction, Repair

## Abstract

We describe a technique to repair ischemic ventricular septal rupture via a left ventriculotomy. It employs a large endoventricular patch as a “lining” over the locally patched septal defect and the free wall defect which is going to be roofed with an external patch. Both defects are then closed in double layers, holding a single continuous patch. The technique enhances the advantage of the left ventriculotomy in the repair and minimizes ventriculotomy-related morbidity.

While the trans-left ventricular approach allows reliable repair of ventricular septal rupture, great care must be taken when closing the ventriculotomy often made at the infarcted part of the left ventricle.

We modified Dr. Daggett’s method specifically designed for the repair of posterior septal rupture [1] by introducing a large endoventricular patch which functions as a single lining over the infarcted area [2]. We have found that the modification is effective not only for repairing posterior septal ruptures but also for anterior septal rupture. A lining patch was versatile enough to work in either position.

## Surgical technique

The heart was exposed through a median sternotomy and cardiopulmonary bypass established with bicaval cannulation and ascending aortic return.

In case of the posterior septal rupture, the apex of the beating heart was retracted cephalad with the aid of traction sutures placed in the bottom of the pericardial sac. On total bypass, the heart was arrested with cold anterograde crystalloid cardioplegia, and then vented via the left upper pulmonary vein.

A left ventriculotomy was made parallel to the descending coronary artery on the interventricular groove. In the posterior aspect, a left ventriculotomy was made closely to the septum, since the attachment of the posterior papillary muscle was even closer to the septum than that of the anterior papillary muscle. Additionally, a small amount of infarcted myocardium was excised from the edges of incision to obtain a larger working space.

With balanced traction on stay sutures placed on bilateral edges of the ventriculotomy, the ventricular septal defect was exposed.

**Fig. 1:**
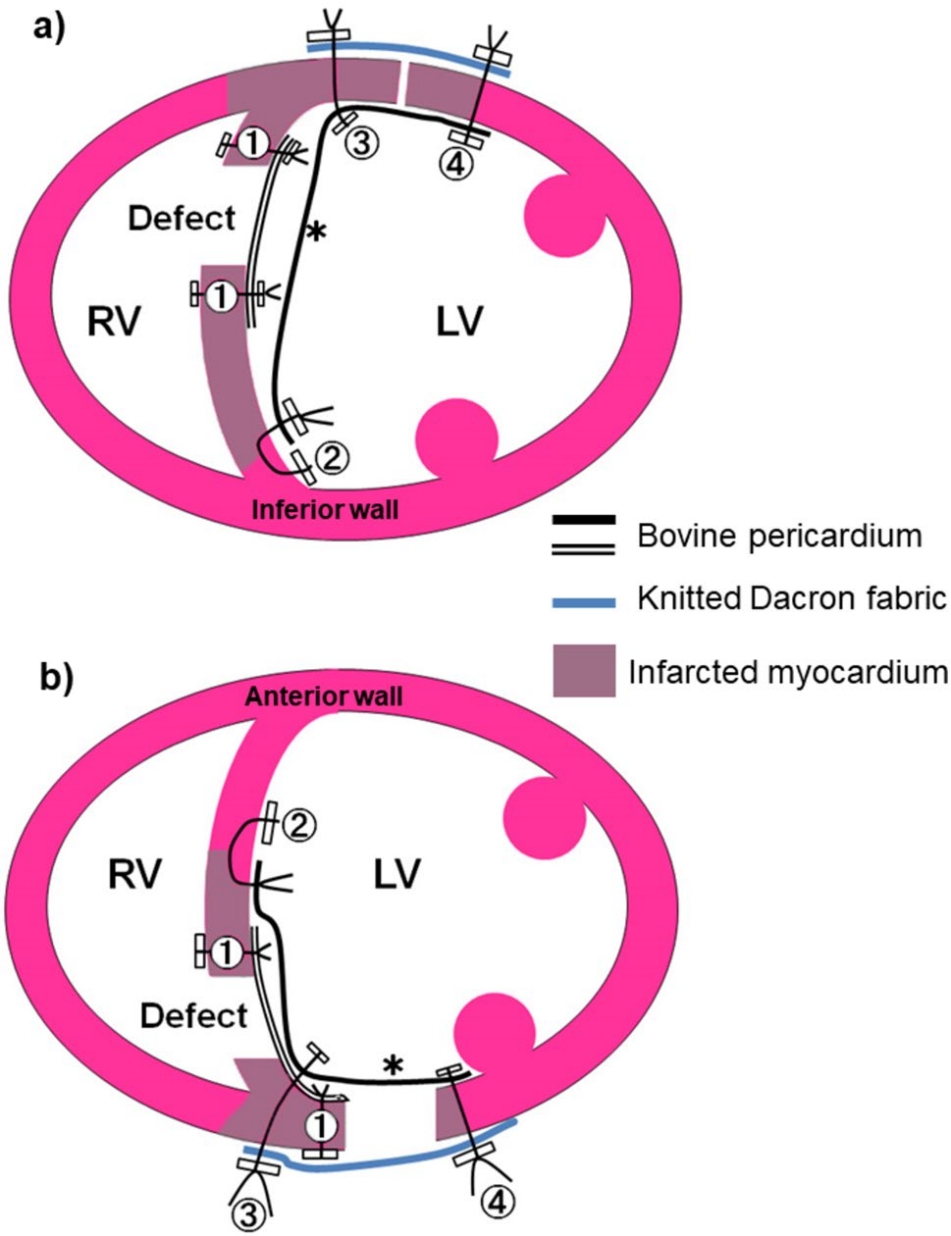
Schematic diagrams of the repair techniques. **a** Repair of anterior septal rupture. **b** Repair of posterior septal rupture. Sutures are numbered and placed in numerical order. A cross section shows the endoventricular “lining” patch (*) closes both the septal and free wall defects separately with the hinge stitches between (③)

**Fig. 2:**
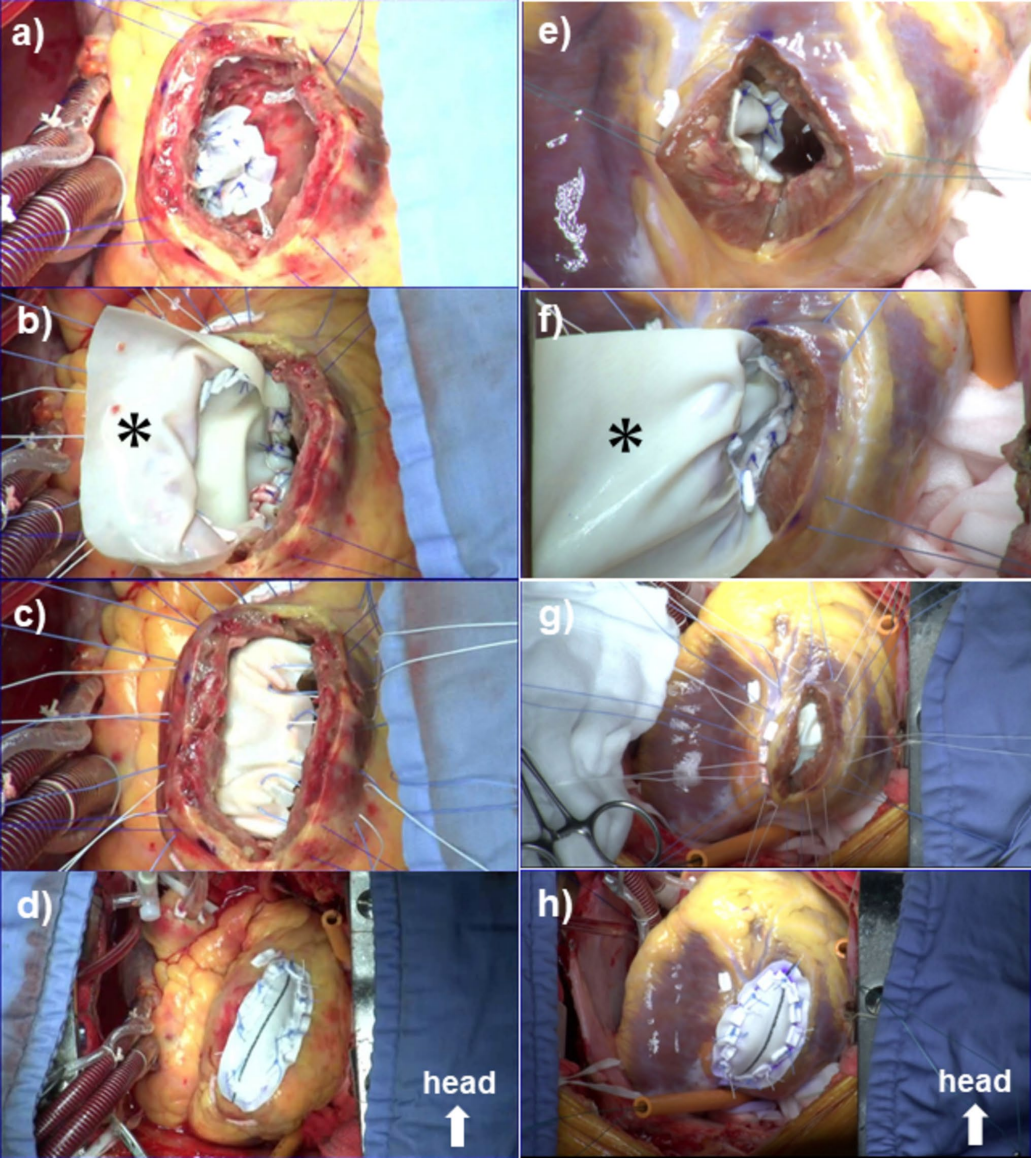
Operative steps of closure of the septal defect and the ventriculotomy. **a**–**d** Repair of anterior septal rupture. **e**–**h** Repair of posterior septal rupture. The diaphragmatic aspect of the heart is shown by lifting the apex towards the head. *The lining patch

Interrupted mattress stitches of 4/0 polypropylene Teflon-pledgeted suture (① in Fig. 1a) were placed around the ventricular septal defect from the right ventricular side into the left ventricle. In case of rupture of the posterior septum, these stitches were continued until the free wall of the ventricle was reached, and then, the direction of the needle was switched to the outside-in fashion on the free wall for technical ease (① in Fig. 1b). An oval bovine pericardial patch was attached to the ventricular septum (Fig. 2a, e).

Interrupted mattress stitches of the same suture were then placed in the non-infarct area of the surrounding ventricular septum (② in Fig. 1a, b). These stitches were given with deep bites and then continued until the free wall of the ventricle was reached. Here, a large patch with a hemi-circular head was fashioned out of the rest of the bovine pericardial sheet. The stitches were then passed through the round head of this patch and secured it on the ventricular septum over the already patched septal defect (Fig. 2b, f).

Interrupted sutures of 2/0 braided synthetics with Teflon pledgets (③ in Fig. 1a) were then placed in a horizontal mattress fashion through the folded edge of the patch and then from the inside of the left ventricle to the outside on the interventricular groove. In the posterior septal repair, the stitches at the middle of this row incorporated the primary patch into bitten layers (③ in Fig. 1b). This row of mattress sutures in combination with the prior endocardial stitches was to complete the secondary closure of the ventricular septal defect.

The remaining portion of the patch was then turned over laterally and fashioned for closing the ventriculotomy as well as lining over the infarcted free wall.

Another set of Teflon-pledgeted interrupted sutures (④ in Fig. 1a, b) were passed through the tail of the patch and then from the inside of the left ventricle to the outside along the left side border of the infarcted myocardium. The base of the posteromedial papillary muscle was well off those stitches.

When all the sutures were in place, the free wall defect created by ventriculotomy was ringed about with 2/0 braided sutures and bottomed in about 10 mm width by the secondary and now “lining” endoventricular patch　(Fig. 2c, g). An appropriately but slightly undersized patch of collagen-sealed, velour knitted Dacron was fashioned to cover the free wall defect. The whole set of sutures secured the prosthesis to the heart with the buttress of Teflon pledgets (Fig. 2d, h).

We employed the technique in three consecutive patients, one with anterior septal rupture and another two with posterior septal rupture. All three patients weaned from cardiopulmonary bypass with moderate inotropic support and the intraaortic balloon pump. There were no additional stitches required for the ventriculotomy site.

Aortic cross-clamp time ranged from 151 to 188 min and pump time ranged from 200 to 262 min.

The first two patients recovered uneventfully and were discharged on the 24th and 20th postoperative day. Echocardiography at hospital discharge revealed mild or less mitral insufficiency but no residual shunt or blood flow in the gap between the double patches. The ejection fractions were 0.43 and 0.55, respectively.

The last patient with posterior septal rupture presented with right ventricular infarction. A postoperative echocardiogram on day 7 showed no interventricular communication. Although the left ventricular ejection fraction increased from 0.34 to 0.45 for the period, the patient expired on the 30th postoperative day from sepsis.

## Comments

Ventricular septal rupture after myocardial infarction is extremely fatal, and there are still many challenges in its surgical repair. Historically, several techniques for surgical repair have been reported, most of which involve applying a large patch to the left side of the ventricular septum through a left ventricular incision [1, 3].

Repairs via the right ventricle have also been reported with comparable results [4], but even in such cases, it is important to place a patch of sufficient size on the left ventricular side of the interventricular septum. Combining the two-layer method or the sandwich method results in more reliable closure of the defect [3, 5].

Our technique takes advantage of the left ventricular incision, which allows placement of a large patch on the left ventricular side, and offsets the disadvantage of potentially fatal bleeding from the ventriculotomy site by restoring ventricular continuity without everting the friable muscle of the infarcted left ventricular free wall. We focused on closing the free wall using a sandwich method with transmural fixation, and noted that by sharing the innermost patch between the septum and the free wall, patch connections could be omitted. Thus, the use of a single lining patch expedited multilayered closure of both the septal and free wall defects. The technique is effective when the left ventricular free wall is extensively infarcted. If the free wall remains viable, non-patch methods may be preferred, as contraction of the lined area may be inhibited.

Infarct exclusion is another option [6–8]. It is simple and applicable to a wide variety of septal ruptures. Unfortunately, we have experienced a case where the pressurized endoventricular patch partially dislodged after infarct exclusion, resulting in tension and bleeding at the repaired ventriculotomy. If a secure suture line for the exclusion patch is not available, our method may also be an option.
